# H_2_ Sensing Response of Flame-Spray-Made Ru/SnO_2_ Thick Films Fabricated from Spin-Coated Nanoparticles

**DOI:** 10.3390/s91108996

**Published:** 2009-11-11

**Authors:** Chaikarn Liewhiran, Nittaya Tamaekong, Anurat Wisitsoraat, Sukon Phanichphant

**Affiliations:** 1 Department of Physics and Materials Science, Faculty of Science, Chiang Mai University, Chiang Mai, 50202, Thailand; E-Mail: chaikarn_l@yahoo.com; 2 Nanoscience Research Laboratory, Department of Chemistry, Faculty of Science, Chiang Mai University, Chiang Mai, 50202, Thailand; E-Mail: doramon_koygy@hotmail.com; 3 National Electronics and Computer Technology Center, Pathumthani, 12120, Thailand; E-Mail: a.wisitsoraat@nectec.or.th

**Keywords:** SnO_2_, ruthenium, flame spray pyrolysis, H_2_ sensor

## Abstract

High specific surface area (*SSA*_BET_: 141.6 m^2^/g) SnO_2_ nanoparticles doped with 0.2–3 wt% Ru were successfully produced in a single step by flame spray pyrolysis (FSP). The phase and crystallite size were analyzed by XRD. The specific surface area (*SSA*_BET_) of the nanoparticles was measured by nitrogen adsorption (BET analysis). As the Ru concentration increased, the *SSA*_BET_ was found to linearly decrease, while the average BET-equivalent particle diameter (*d*_BET_) increased. FSP yielded small Ru particles attached to the surface of the supporting SnO_2_ nanoparticles, indicating a high *SSA*_BET_. The morphology and accurate size of the primary particles were further investigated by TEM. The crystallite sizes of the spherical, hexagonal, and rectangular SnO_2_ particles were in the range of 3–10 nm. SnO_2_ nanorods were found to range from 3–5 nm in width and 5–20 nm in length. Sensing films were prepared by the spin coating technique. The gas sensing of H_2_ (500–10,000 ppm) was studied at the operating temperatures ranging from 200–350 °C in presence of dry air. After the sensing tests, the morphology and the cross-section of sensing film were analyzed by SEM and EDS analyses. The 0.2%Ru-dispersed on SnO_2_ sensing film showed the highest sensitivity and a very fast response time (6 s) compared to a pure SnO_2_ sensing film, with a highest H_2_ concentration of 1 vol% at 350 °C and a low H_2_ detection limit of 500 ppm at 200 °C.

## 1. Introduction

SnO_2_ is one of the most promising materials for sensors and it has attracted the attention of scientists interested in gas sensing applications under atmospheric conditions. Semiconducting metal oxides in general, and SnO_2_ in particular, have been investigated extensively for the purpose of practical applications such as gas leak detecting and environmental monitoring. It is a wide band gap (3.6 eV) n-type semiconductor and the best-understood prototype of oxide-based gas sensors for the detection of reducing gases (like CO [1-6], H_2_ [6-12], SO_2_ [13,14], NH_3_ [15,16], H_2_S [11,17], C_2_H_5_OH [18]) or oxidizing gases (like NO_2_ [1,5,12], O_2_ [19,20]) in air. The detection of H_2_ gas in different industrial applications is especially important for safety reasons. The development of a gas sensor for 10–10,000 ppm of H_2_ gas is also of high interest since H_2_ is one of the main gases evolving under pyrolysis in the initial stage of combustion. H_2_ gas leaks easily from gas lines and systems and is one of the most explosive gases.

The electrical properties of nanocrystalline SnO_2_ strongly depend on crystallite size and surface state produced by gas adsorption which results in the space charge appearance and band modulation [5]. The flame aerosol synthesis method is one of the most promising routes for the formation of single and multi-component functional nanoparticles at low cost and high production rate from gases in a flame. The sizes of the particles range from a few to several hundred nanometers in diameter, depending on the material and process conditions. The FSP process was systematically investigated using an external-mixing gas-assisted atomizer supported by premixed methane and oxygen flamelets [21-23]. In flame reactors, the energy of the flame is used to drive chemical reactions of precursors resulting in clusters which further grow to nanoparticles by surface growth and/or coagulation and coalescence at high temperatures. Therefore, the FSP is a very promising technique for sensor material fabrication since it enables primary particle and crystal size control [21-24], which are important to improve the sensitivity, as well as the controlled *in situ* deposition of noble metal clusters [2]. FSP also has the advantage of allowing one to completely manufacture the nanopowder in a single high-temperature step without affecting the microstructure and noble metal particle size in a subsequent annealing process [25]. Moreover, the importance of the size control, the required large and easily accessible surface area (large pore size, no micropores) the desired high crystallinity, the efficiency of noble metal doping (i.e., Pt, Pd, and Ru) and competitive production rates put high demands on any chosen method of nanoparticle production for sensor materials.

The gas sensors based on SnO_2_ and metal-doped SnO_2_ nanostructures were found to be good candidates for detecting both reducing and oxidizing gases of various concentrations. Many researchers have reported that pure SnO_2_ and metal-doped SnO_2_ could be widely used to detect H_2_ vapor [6-12]. A summary comparing gas sensing with pure SnO_2_ and metal-doped SnO_2_ prepared by several synthetic methods is shown in Table 1. The effect of catalytic Ru doping, as well as the sensing temperature, on the sensor characteristics of sensing films were reported. It has been shown that the sensor characteristics of sensing films are affected by the particles morphology, Ru doping levels, and the operating temperatures, are all important parameters that affect the gas sensing properties in terms of high sensitivity, fast response and recovery time. FSP and spin coating technique have several advantages in producing the nano-sized particles and regular sensing films suitable for the gas sensor. Especially, the Ru additives increase the rate of specific reactions on the surface of SnO_2_ grain due to spill-over effect of modification of surface energy states. Also, Ru metals were intentionally introduced for certain gases, promoting the receptor function and thus improving the sensing behaviors in terms of the selectivity and time factors. Therefore in the present study, it was of interest to apply FSP for a new production of Ru/SnO_2_ nanoparticles for use as H_2_ gas sensor.

## 2. Experimental

### 2.1. Flame Synthesis of Nanopowders

**Figure 1.**
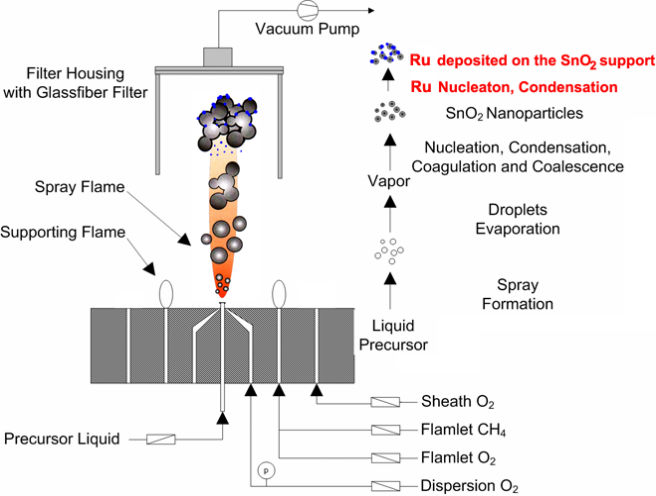
Schematic of the FSP experimental set up for the synthesis of samples P0-P3.

**Figure 2.**
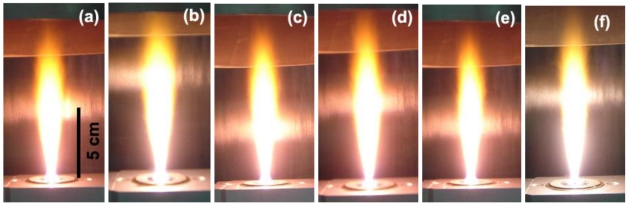
Spray flame of: (a) pure SnO_2_, (b–f) 0.2–3 wt% Ru/SnO_2_ nanoparticles producing 5 ml/min of liquid precursor feed rate and dispersed by O_2_ (5 l/min) at 1.5 bar pressure drop across the nozzle tip. The flame heights were observed ranging from 10–12 cm with slight increasing the combustion enthalpy and Ru concentrations.

The experimental setup for the synthesis of pure SnO_2_, 0.2–3 wt%Ru/SnO_2_ nanopowders by FSP is shown in Figure 1. The flame-spray-made (5/5) pure SnO_2_ was designated as P0 while the SnO_2_ nanopowders doped with 0.2, 0.6, 1, 2, and 3 wt%Ru were designated as P0.2, P0.6, P1, P2, and P3, respectively. Precursor solutions (0.5 M) were prepared by dissolving appropriate amounts of tin (II) 2-ethylhexanoate (Aldrich, 95%) and ruthenium (III) acetylacetonate (Aldrich, 97%) used as Sn and Ru precursors in xylene (Carlo Erba, 98.5%), respectively. In a typical run, the precursor mixture was fed into a nozzle at a constant feed rate of 5 mL/min using a syringe pump. At the end of the nozzle the precursor solution was dispersed by 4.30 L/min oxygen forming a spray with a pressure drop at the capillary tip kept constant at 1.5 bars by adjusting the orifice gap area. A sheath gas flow of 3.92 L/min of O_2_ was issued concentrically around the nozzle to stabilize and contain the spray flame. The spray was ignited by supporting flamelets fed with oxygen (2.46 L/min) and methane (1.19 L/min) which are positioned in a ring around the nozzle outlet. The observed flame height was approximately 10-12 cm, and it increased slightly with increasing combustion enthalpy. The combustion enthalpies are directly dependent on the particular solvent, starting materials, and dopants used. Pure SnO_2_ samples show an light orange and Ru doped samples show light pink color in the base and middle of the flame, and also light orange on the top of the flame, as shown in Figure 2. After evaporation and combustion of precursor droplets, particles are formed by nucleation, condensation, coagulation, coalescence, and Ru deposit on the SnO_2_ support. Finally, the nanoparticles were collected on a glass microfibre filters (Whatmann GF/A, 25.7 cm in diameter) with the aid of a vacuum pump (Busch, Seco SV 1040C).

### 2.2. Powder Characterizations

The powder phases were analyzed by X-ray diffraction (XRD) [Phillips X-′pert] using CuKα radiation (20 kV, 20 mA) with a scanning speed of 5°/minute. The specific surface areas of the nanopowders were obtained from BET measurements [Autosorb 1 MP, Quantachrome]. All samples were degassed at 120 °C for 2 h prior to analysis. The diameter of particles were calculated from *d*_BET_ = 6/*SSA*_BET_ × *ρ*_sample_, where *SSA*_BET_ is the specific surface area (m^2^/g), *ρ*_samples_ are the average density of SnO_2_ (*ρ*_SnO2_ = 6.85 g/cm^3^ [1]) and the density of ruthenium (*ρ*_Ru_ = 10.65 g/cm^3^ taken into account for their weight content of different doping [26]). The accurate morphologies of the nanoparticles and cross-section structures of sensor were analyzed by TEM [JSM-2010, JEOL], SEM [JSM-6335F, JEOL], and EDS analyses.

### 2.3. Paste and Sensor Preparations

An appropriate quantity of 0.28 mL homogeneous mixed solution was prepared by stirring and heating at 80 °C for 12 hr with ethyl cellulose (Fluka, 30–70 mPa.s) as the temporary binder and terpineol (Aldrich, 90%) as a solvent. The liquid mixture was combined with 60 mg samples of the P0, P0.2, P1, and P3 nanopowders and mixed for 30 min to form a paste prior to spin-coating. The resulting paste was firstly spin-coated at 700 rpm for 10 s, and then subsequently at 3,000 rpm for 30 s on the Al_2_O_3_ substrates interdigitated with Au electrodes (0.5 × 0.5 cm) to deposit sensing films. The resulting substrates were annealed in an oven at 150 °C for 1 h with an annealing rate of 1 °C/min and at 400 °C for 1h with an annealing rate of 1 °C/min for binder removal prior to the sensing test [28].

### 2.4. Sensor Measurement

The sensor characteristics of sensing films were characterized toward the high concentration of H_2_ gas (500–10,000 ppm). The flow through technique was used to test the gas-sensing properties of sensing films. A constant flux of synthetic air of 2 L/min as gas carrier was flown to mix with the desired concentration of pollutants dispersed in synthetic air. All measurements were conducted in a temperature-stabilized sealed chamber at 20 °C under controlled humidity. The gas flow rates were precisely manipulated using a computer controlled multi-channel mass flow controller. The external NiCr heater was heated by a regulated DC power supply to different operating temperatures. The operating temperature was varied from 200 °C to 350 °C. The resistances of various sensors were continuously monitored with a computer-controlled system by voltage-amperometric technique with 10 V DC bias and current measurement through a picoammeter. The sensor was exposed to a gas sample for ∼5 minutes for each gas concentration testing and then the air flux was restored for 15 minutes. The sensitivity (*S*) is defined in the following as the resistance ratio *R*_a_/*R*_g_ [11,27-30], where *R*_a_ is the resistance in dry air, and *R*_g_ is the resistance in the test gas. The response time (*T*_res_) is defined as the time required until 90% of the response signal is reached. The recovery time (*T*_rec_) denotes the time needed until 90% of the original baseline signal is recovered. After the sensors fabricated using samples P0, P0.2, P1, and P3 had been tested with varied the operating temperatures, they were designated as S0, S0.2, S1, and S3, respectively. Finally, the morphologies, film thickness of sensing layers and elemental compositions were further analyzed by SEM and EDS line-scan mode analyses.

## 3. Results and Discussion

### 3.1. Nanopowder Properties

**Figure 3.**
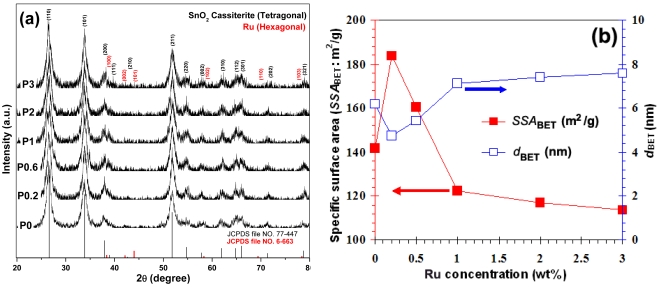
(a) XRD and (b) BET data of flame-made (5/5) 0–3 wt%Ru/SnO_2_ as-prepared (P0-P3).

Figure 3(a) shows the XRD patterns of flame-spray-made pure SnO_2_ and 0.2–3 wt%Pd/SnO_2_ nanopowders. All samples were highly crystalline, and all peaks can be confirmed to be the cassiterite-tetragonal phase of SnO_2_, which matched well with the JCPDS file No. 77-447. Ru peaks were not found in these patterns (JCPDS file No. 6-663). It can be assumed that the amount of Ru concentration was very low, which affected the appearance of the Ru peaks.

**Figure 4.**
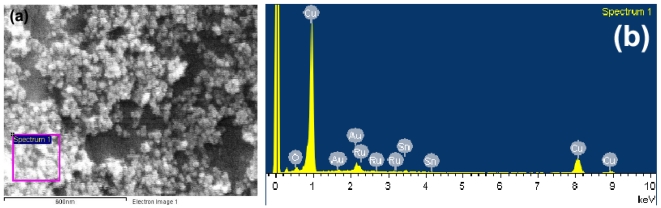
(a) SEM micrographs of P3 samples. The EDS spectra for the square region indicated in (b) P3 sample contain Ru deposited on SnO_2_ support spin-coated on the Au/Al_2_O_3_ substrate.

The diffraction peak for 0.2 wt% Ru/SnO_2_ nanopowder was the broadest compared to other doping levels, suggesting relatively well-dispersed smaller Ru particles. As the Ru concentration increased, all peaks were slightly sharpened and increased in intensity, indicating that the poor-dispersion of larger Ru particles leads to rough agglomeration at higher Ru doping levels. These results were consistent with the BET data, as shown in Figure 3(b). The specific surface area (*SSA*_BET_) drastically increased from 141.6 m^2^/g (bare SnO_2_) to 183.8 m^2^/g (0.2 wt%Ru/SnO_2_). When the Ru concentration increased (0.2 to 3 wt%Ru), the *SSA*_BET_ were found to linearly decrease (183.8 to 113.5 m^2^/g), with an increase in the average BET-equivalent particle diameter (*d*_BET_) (bare SnO_2_: 6.2 nm, 0.2–3 wt%Ru/SnO_2_: 4.7 to 7.6 nm). This trend was consistent with Niranjan *et al.* [11] who studied the effect of Ru concentration on crystalline SnO_2_ nanoparticles. To explain this result, it can be speculated as follows: during the processes of Ru particle formation and deposition on the particle support (SnO_2_) in the flame, the Ru created a new nucleation center, which in turn changed the nucleation type from homogeneous to heterogeneous, and deteriorated the deposition formation leading to the agglomeration of the tiny Ru particles at high doping levels. This can be confirmed from the accurate morphology by TEM bright-field images. The FSP afforded small Ru particles attached to the surface of the supporting SnO_2_ nanoparticles indicating a high *SSA*_BET_. The well-dispersed flame-made 0.2 wt%Ru/SnO_2_ nanoparticles were confined to the SnO_2_ surface. The larger crystallite diameters indicate clumping and clusters of Ru, translating into a poor dispersion of the Ru nanoparticles on SnO_2_ support which affected to the decrease of the *SSA*_BET_. The SEM micrograph [Figure 4(a)] and the elemental compositions of the agglomerated nanoparticles formed with the sample with the highest Ru concentration (P3) are shown by the EDS spectra in Figure 4(b). Interestingly, the analyzed square regions [Figure 4(b)] were composed of the agglomerated nanoparticles, the copper grid, and gold sputtering prior to an analysis. The EDS spectra showed elemental compositions rich in copper (Cu), caused by the contamination of copper foil, poor gold (Au) caused by the contamination of gold sputtering which used to prepared the samples prior to an analyzing, tin (Sn), oxygen (O), and poor ruthenium (Ru) elements.

**Figure 5.**
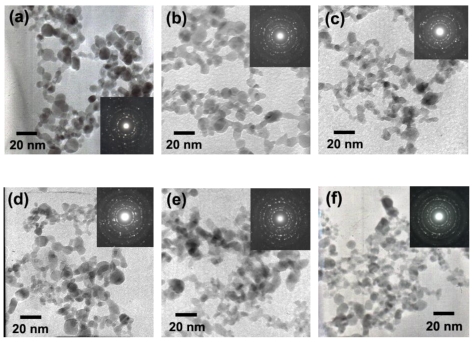
(a) shows TEM bright-field images of highly crystalline flame-made (5/5) SnO_2_ nanoparticles (P0) and (b–f) 0.2–3 wt%Ru/SnO_2_ nanoparticles (P0.2-P3) with the same magnifications. Insets show the corresponding diffraction patterns of the nanoparticles.

Figure 5 (a–f) show TEM bright-field images of P0-P3. The corresponding diffraction patterns are shown in the insets. The diffraction patterns illustrated spot patterns corresponding to the tetragonal-cassiterite structure of SnO_2_, indicating the SnO_2_ nanoparticles were highly crystalline, in good agreement with the XRD data. The TEM bright-field images of the FSP (5/5)-made nanoparticles, indicate polyhedral aggregates of primary particles. The morphologies of flame made (5/5) SnO_2_ and 0.2–3 wt% Ru/SnO_2_ nanoparticles contained mainly spherical particles, with diameters ranging from 3–10 nm, with occasional rectangular, hexagonal (3–10 nm) and rod-like (3–5 nm in width, and 5–20 nm in length) particles. Ru nanoparticles were not found in these micrographs. This is because Ru is very small when compared with the size of SnO_2_ nano-support. The primary particle diameters observed by TEM were consistent with the *d*_BET_. From these data, it can be clearly seen that the amount of Ru concentrations would not affect to change the size of SnO_2_ nanoparticles. We could assume this doping formation from the *Hume-Rothery* rules [31-33], which commonly used to explain the solid mixtures called solid solutions.

In the doping of materials, atoms of the solvent (host material; Sn) are successfully replaced by the solute-atoms (the doping atom; Ru) from their lattice positions (interstitial solid solutions are not discussed here). In the other words, one material gets dissolved in the other, without disturbing the crystal structure, except for lattice distortions (expansions or compressions). For the formation of solid solutions, according to the *Hume-Rothery* rules, some criteria have to be fulfilled: (1) the atomic radii of the solute (Ru = 178 pm) [34] and solvent (Sn = 145 pm) [34] atoms must differ by no more than 15% (∼22.75%). If not, it is likely to have a low solubility. This is the first rule which must be considered. The atomic size factor was said to be unfavorable; (2) the solute and solvent should have similar electronegativity (Ru = 2.2, Sn = 1.8) [33], compared to the host. If the electronegativity difference is too great, the metals will tend to form intermetallic compounds instead of solid solutions. Its solubility in the host would therefore be limited, because of the so-called electronegative valency effect; (3) a metal with lower valency is more likely to dissolve one which has a higher valency, than *vice versa* (relative valency effect). The valence electrons are the electrons in the last shell or energy level of an atom. Maximum solubility occurs when the solvent (Sn) [35] and solute (Ru) [36,37] have the same valency. Moreover, the thermodynamic instability of the lower oxidation states of Ru was discussed by Wiley *et al.* [36-38] to explain their inability to synthesize oxygen deficient Ru-bearing perovskites for catalysis. Although spectroscopic data indicated that other oxidation states (Ru^2+^, Ru^3+^, Ru^5+^) could exist in oxides, species other than Ru^4+^ generally occurred in mixed-valence phases dominated by Ru^4+^. Exceptions exist, however, and Ru^5+^ and even Ru^7+^ occurred in oxide compounds where there were essential structural constituents and the only Ru species. For this reason, a more precise generalization that Ru^4+^ was the lowest valence in oxides which was not induced by the special defect equilibrium. Metals with lower valency will tend to dissolve metals with higher valency; and (4) the crystal structures of solute (Ru = hexagonal) and solvent (Sn = tetragonal) must match. Thus the size of particles in the doped sample were not affected by Ru due to the fact that Ru could not get in solid solution into the unit cell of SnO_2_ crystal structure.

### 3.2. Gas Sensing Properties

**Figure 6.**
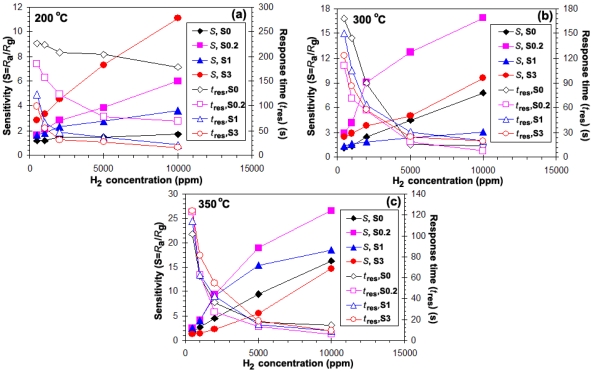
(a–c) Sensing performance in the terms of the sensitivity (filled symbols, left axis) and corresponding response times (open symbols, right axis) of pure SnO_2_ (S0) sensors and doped with 0.2, 1, 3 wt% (S0.2, S1, S3) sensors as a function of H_2_ concentration in dry air at (a) 200, (b) 300, and (c) 350 °C. The sensitivity increased and the response times decreased with increasing H_2_ concentration.

**Figure 7.**
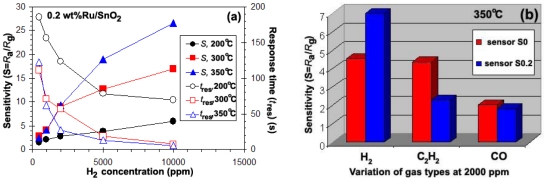
(a) Sensing performance in the terms of the sensitivity (filled symbols, left axis) and corresponding response times (open symbols, right axis) of 0.2 wt%Ru/SnO_2_ (S0.2) sensors as a function of H_2_ concentration in dry air at 200 °C (circles), 300 °C (rectangles), and 350 °C (triangles). The sensitivity increased and the response times decreased with increasing H_2_ concentration and operating temperature. (b) Selectivity histogram of pure SnO_2_ and containing 0.2 wt%Ru for different gases (0.2%vol) at 350 °C.

Figures 6(a-c) show the plot of sensitivity (*S*) and response times (*T*_res_) versus hydrogen concentrations ranging from 500–10,000 ppm for the sensors S0, S0.2, S1, and S3 during a forward cycle at operating temperature ranging from 200–350 °C. It was found that the sensitivity increased with operating temperature to the maximum at 350 °C [Figure 6(c)]. Interestingly, the temperature of maximum sensitivity was found to shift towards lower Ru concentrations, which can be attributed to the effect of particles size and the specific surface area, as a result of a well-dispersed Ru incorporation into the SnO_2_ matrix. At the operating temperature of 200 °C, the sensitivity of all Ru doping materials was seen to be higher than that of pure SnO_2_. The sensitivity (filled symbols, left axis) increased and the response time (open symbols, right axis) decreased with increasing H_2_ concentrations. Moreover, it was found that the highest Ru concentration (3 wt%) showed the best sensing performance in terms of sensitivity (*S* = 8.6) and response time. The response time of a 3 wt% Ru/SnO_2_ sensor for 10,000 ppm at 200 °C was 16 s (open circles, right axis), which was better than pure SnO_2_ (178 s) (open diamonds, right axis) and the other doping levels (0.2 wt% Ru/SnO_2_ = 70 s (open rectangles, right axis), and 1 wt% Ru/SnO_2_ = 22 s (open triangles, right axis)). On the other hand, both the operating temperatures of 300 °C and 350 °C had better sensing performance than 200 °C in terms of sensitivity (filled symbols, left axis) and faster response time (open symbols, right axis). Also, in the case of Ru doping the best performance was achieved at a sensor operating temperature of 350 °C. However, the situation was completely different when more Ru was added. Here, 1 wt% (300 °C) and 3 wt% Ru/SnO_2_ (350 °C) also displayed evidently reduced sensing performance in terms of sensitivity. Note that these tests were performed with a set of four sensors placed in the chamber. The sensitivities of all sensors were found to increase rather linearly with increasing H_2_ concentrations. As the Ru concentration increased from 0.2 to 3 wt%Ru, the lowest Ru concentration (S0.2) the sensor behaviors improved in terms of the best sensitivity (to 10,000 ppm, *S* = 27) (filled rectangles, left axis) and very fast response times (*T*_res_ = 6 s) (open rectangles, right axis) at 350 °C, which evidently were better than S1, and S3. The sensor S0.2 showed very fast response to H_2_ gas, whereas the response of the pure SnO_2_ sensor (S0) was somewhat sluggish. Figures 6(b) and 6(c) indicate the dependence of the sensitivity on the Ru concentration at an operating temperature of 300 °C and 350 °C, respectively. The amount and distribution of Ru species in the SnO_2_ support were important parameters governing the sensitivity, being maximum (S = 27) at 350 °C for a SnO_2_ containing 0.2 wt% Ru. The sensitivity consistently increased with increasing H_2_ concentration. The role of the Ru in enhancing the sensitivity and response rate of the sensor could be due to the electronic interaction between the sensitizer and the semi-conducting material. Ru acts as a catalyst and enhances the reaction rate, especially because χ_o_ − χ_Ru_ < χ_o_ − χ_Sn,_ where χ_o_ represents the electronegativity value (χ_o_, χ_Sn_, χ_Ru_ = 3.5, 1.8, 2.2, respectively) [11]. Thus, when oxygen is adsorbed on the Ru zones of strong localization at elevated temperatures, the potential between the SnO_2_ grains may be raised and as a result, the total resistance increases as compared with the sample without Ru. The decrease of the amount of Ru concentration leads to well-dispersed Ru on the SnO_2_ surface arising from the chemisorbed oxygen species. Moreover, Figure 7(a) shows the response to high concentrations of H_2_ (500–10,000 ppm) of sensors which were functionalized *in situ* with 0.2 wt% Ru. Doping the SnO_2_ with 0.2 wt% Ru results in a much steeper calibration curve and the highest sensor signal compared to pure SnO_2_ [see Figure 7(a)]. The higher sensor signal, and especially the higher sensitivity (i.e., the steeper response curve), demonstrate an enhanced sensor performance.

Figure 6 shows the selectivity histogram for 0.2 vol% of different gases at an operating temperature of 350 °C. The sensors S0 and S0.2 exhibited similar selectivity towards the flammable H_2_ and C_2_H_2_ gases and toxic CO gas. This can be attributed to the identical reducing behavior of both gas types. The S0.2 sensor has a good gas selectivity for 0.2 vol% H_2_ of 7 at 350 °C. The sensitivity of S0.2 sensor of C_2_H_2_ and CO gases were 2.3 and 1.8 at 0.2 vol% H_2_ at 350 °C. Thus, the gas sensitivity of S0.2 sensor was higher than that of C_2_H_2_ and CO gases. The H_2_ selectivity of S0.2 sensor was substantially higher compared to pure SnO_2_ gas sensor (S0). On the other hand, C_2_H_2_ and CO gases sensitivity/selectivity of S0.2 sensor was also evidently deteriorated compared to that of pure SnO_2_ gas sensor (S0). Ru cannot improve the sensing performance and is unsuitable for use as dopant in SnO_2_ sensor for both C_2_H_2_ and CO gases. This is because the absorption configurations of the gas molecules and the surface fragmentation reactions on the Ru sites are responsible for the similar sensitivity values towards all gases.

### 3.3. SEM-Film Thickness Sensing Layer

**Figure 8.**
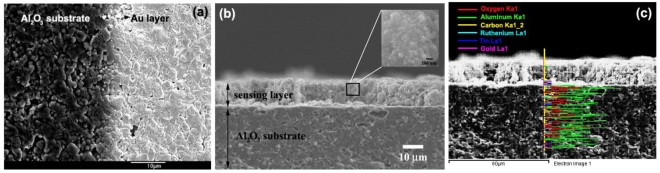
(a) the phase boundaries of microstructures of high density Al_2_O_3_ (dark view, left) substrate interdigitated with Au electrodes (bright view, right), (b) The film thickness was approximately 10 μm (P0.2) spin-coated onto Au/Al_2_O_3_ substrate (side view) cross-section after sensing at 350 °C in dry air (S0.2). The square emphasizes how the particle sizes are slightly changed after the annealing and sensing test was also shown in the inset and (c) EDS elemental-line scan analysis.

The microstructures of high density Al_2_O_3_ (dark view) substrate interdigitated with Au electrodes (bright view) was evidently seen as the phase boundaries in Figure 8(a). The cross-section, film thickness, and surface morphology of the sensing film layer (S0.2) after a sensing test at 200–350 °C were observed using SEM analysis as shown in Figure 8(b). The film thickness of sensing film was about 10 μm, which was of tremendous benefit to the H_2_ gas sensing properties. The microstructure of high density Al_2_O_3_ substrate was visible. The square emphasized the investigation selected area at high magnification to an aggregated of primary particles after sensing test. The particle sizes of nanoparticles slightly changed after annealing and sensing test were also shown in the inset. In addition, the trends in the elemental composition of the agglomerated nanoparticles formed of sample P0.2 was shown by the EDS line scan mode in Figure 8(c). Interestingly, the analyzed regions composed of the nanoparticles, the copper grid, and gold sputtering prior to an analysis. The line scan across the agglomerate for sensor P0.2 is indicated in Figure 8(c). The elemental-line histograms are shown as a series of solid lines corresponding to a rich in copper (Cu) caused by the contamination of copper grid, poor gold (Au), tin (Sn), oxygen (O), and ruthenium (Ru) elements. After annealing process, a denser film layer was formed. Regularities and preciseness in the film thickness stem from the spin coating technique.

## 4. Conclusions

FSP was successfully performed for the synthesis of pristine SnO_2_ and 0.2–3 wt% Ru/SnO_2_ nanopowders for a H_2_ gas sensing application. The effect of Ru content on the doping of SnO_2_ nanoparticles can be assumed according to the *Hume-Rothery* rules. It was noticed that the Ru could not form into the crystal structure of SnO_2_ in solid solution, thus the size of particles in the doped samples were not affected by the Ru atoms. The fabricated sensors were prepared by the spin coating technique. It can be concluded that the highest sensitivity and very fast responses to H_2_ gas were obtained by the incorporation at the lowest concentration of Ru (0.2 wt%) and the highest operating temperature (350 °C). The response time was within 6 s for 1 vol% H_2_ in presence of dry air. The 0.2 wt%Ru/SnO_2_ sensor has good gas selectivity for 0.2 vol% H_2_ at 350 °C.

## Figures and Tables

**Table 1.** Summary on comparison of metal-doped SnO_2_ with several methods for gas sensing.

**Authors**	**Method**	**Doping level**	**Gas Concentration**	**Sensing performances**
Sahm *et al.* [1]	FSP (nanopowders) Drop coating (sensors)	Pure SnO_2_	NO_2_ (10–5,000 ppb), CO (500–10,000 ppm), propanol (10–300 ppm)	NO_2_; Sensitivity: ∼20 to 5,000 ppb at 220 °C
				Propanal; Sensitivity: ∼300 to 150 ppm at 220 °C
Mädler *et al.* [2]	FSP (nanopowders) Thermophoretic deposition (sensors)	0.2 wt%Pt/SnO_2_	CO; 50 ppm	Sensitivity: 8 to 50 ppm at 350 °C
Salehi [9]	Evaporation, Chemical Vapor Deposition, Spray Pyrolysis, Sputtering	In/SnO_2_	H_2_; 500–3,000 ppm	Response to 7% H_2_ at 200 °C of 0.5 s
Ryzhikov *et al.* [10]	Magnetron Sputtering: Sensing film; Laser Ablation: Doping process	Pt/SnO_2_	H_2_; 20–20,000 ppm	Sensitivity: 630 to 1,000 ppm at 300 °C
Niranjan *et al.* [11]	Modified Pechini Route	0.2–0.7 wt%Ru/SnO_2_	H_2_; 700 vol ppm	0.6 wt%Ru/SnO_2_ Sensitivity: 150 at 275 °C
				Response time: 3 s at 275 °C
				Recovery time: 5–10 min at 275 °C

## References

[1] Sahm T, Mädler L., Gurlo A., Barsan N., Pratsinis S.E., Weimar U. (2004). Flame spray synthesis of tin dioxide nanoparticles for gas sensing. Sens. Actuat. B-Chem..

[2] Mädler L., Roessler A., Pratsinis S.E., Sahm T., Gurlo A., Barsan N., Weimar U. (2005). Direct formation of highly porous gas-sensing films by in situ thermophoretic deposition of flame-made Pt/SnO_2_ nanoparticles. Sens. Actuat. B-Chem..

[3] Mädler L., Sahm T., Gurlo A., Grunwaldt J.-D., Barsan N., Weimar U., Pratsinis S.E. (2006). Sensing low concentrations of CO using flame-spray-made Pt/SnO_2_ nanoparticles. J. Nanopart. Res..

[4] Ponce M.A., Castro M.S., Aldao C.M. (2006). Resistance and capacitance analysis of Pd-doped and undoped SnO_2_ thick films sensors exposed to CO atmospheres. Ceram. Inter..

[5] Safonova O.V., Rumyantseva M.N., Ryabova L.I., Labeau M., Delabouglise G., Gaskov A.M. (2001). Effect of combined Pd and Cu doping on microstructure, electrical and gas sensor properties of nanocrystalline tin dioxide. Mater. Sci. Eng..

[6] Baik N.S., Sakai G., Miura N., Yamazoe N. (2000). Hydrothermally treated sol solution of tin oxide for thin-film gas sensor. Sens. Actuat. B-Chem..

[7] Katsuki A., Fukui K. (1998). H_2_ selective gas sensor based on SnO_2_. Sens. Actuat. B-Chem..

[8] Chi-Hwan H., Sang-Do H., Singh I., Toupance T. (2005). Micro-bead of nano-crystalline F-doped SnO_2_ as a sensitive hydrogen gas sensor H_2_ selective gas sensor based on SnO_2_. Sens. Actuat. B-Chem..

[9] Salehi A. (2002). Selectivity enhancement of indium-doped SnO_2_ gas sensors. Thin Solid Films..

[10] Ryzhikov A.S., Shatokhin A.N., Putilin F.N., Rumyantseva M.N., Gaskov A.M., Labeau M. (2005). Hydrogen sensitivity of SnO_2_ thin films doped with Pt by laser ablation. Surf. Coat. Technol..

[11] Niranjan R.S., Hwang Y.K., Kim D.-K., Jhung S.H., Chang J.-S., Mulla I.S. (2005). Nanostructured tin oxide: Synthesis and gas-sensing properties. Mater. Chem. Phys..

[12] Hyodo T., Sasahara K., Shimizu Y., Egashira M. (2005). Preparation of macroporous SnO_2_ films using PMMA microspheres and their sensing properties to NO_x_ and H_2_. Sens. Actuat. B-Chem..

[13] Bukun N, Vinokurov A., Vinokurova M., Derlyukova L., Dobrovolsky Y., Levchenko A. (2005). Chemisorption and electrochemical reactions of SO_2_ on modified SnO_2_ electrodes. Sens. Actuat. B-Chem..

[14] Lančok J., Santoni A., Penza M., Loreti S., Menicucci I., Minarini C., Jelinek M. (2005). Tin oxide thin films prepared by laser-assisted metal-organic CVD: Structural and gas sensing properties. Surf. Coat. Technol..

[15] Wang Y.D., Wu X.H., Su Q., Li Y.F., Zhou Z.L. (2001). Ammonia-sensing characteristics of Pt and SiO_2_ doped SnO_2_ materials. Solid-state Elec..

[16] Teeramonglonrasmee A., Sriyudthsak M. (2000). Methanol and ammonia sensing characteristics of sol-gel derived thin film gas sensor. Sens. Actuat. B-Chem..

[17] Jin C., Yamazaki T., Ito K., Kikuta T., Nakatani N. (2006). H_2_S sensing properties of porous SnO_2_ sputerred films coated with various doping films. Vacuum..

[18] Rella R., Serra A., Siciliano P., Vasanelli L., De G., Licciulli A., Quirini A. (1997). Tin oxide-based gas sensors prepared by the sol-gel process. Sens. Actuat. B-Chem..

[19] Tiburcio-Silver A., Sánchez-Juárez A. (2004). SnO_2_:Ga thin films as oxygen gas sensor. Mater. Sci. Eng..

[20] Maffeïs T.G.G., Owen G.T., Penny M.W., Starke T.K.H., Clark S.A., Ferkel H., Wilks S.P. (2002). Nano-crystalline SnO_2_ gas sensor response to O_2_ and CH_4_ at elevated temperature investigated by XPS. Surf. Sci..

[21] Pratsinis S.E. (1998). Flame aerosol synthesis of ceramic powders. Prog. Energ. Combust..

[22] Mädler L., Kammler H.K., Mueller R., Pratsinis S.E. (2002). Controlled synthesis of nanostructured particles by flame spray pyrolysis. J. Aeros. Sci..

[23] Mueller R., Mädler L., Pratsinis S.E. (2003). Nanoparticle synthesis at high production rates by flame spray pyrolysis. Chem. Eng. Sci..

[24] Mädler L., Stark W.J., Pratsinis S.E. (2002). Rapid synthesis of stable ZnO quantum dots. J. Appl. Phys..

[25] Kammler H.K., Mädler L., Pratsinis S.E. (2001). Flame synthesis of nanoparticles. Chem. Eng. Tech..

[26] Lide D. R. (2006). Properties of the elements and inorganic compounds. CRC Handbook of Chemistry and Physics..

[27] Liewhiran C., Phanichphant S. (2007). Influence of thickness on ethanol sensing characteristics of doctor-bladed thick film from flame-made ZnO nanoparticles. Sensors..

[28] Liewhiran C., Phanichphant S. (2007). Improvement of flame-made nanoparticulate thick film morphology for ethanol sensing. Sensors..

[29] Liewhiran C., Phanichphant S. (2007). Effects of palladium loading on the response of a thick film flame-made zno gas sensor for detection of ethanol vapor. Sensors..

[30] Liewhiran C., Phanichphant S. (2008). Doctor-bladed thick films of flame-made Pd/ZnO nanoparticles for ethanol sensing. Curr. Appl. Phys..

[31] Takeuchi T., Sato H., Mizutani U. (2002). Investigation of Hume-Rothery stabilization mechanism from an initio band calculation for different electron compounds: Cu_5_Zn_8_ and Al-Mg-Zn, Al-Cu-Ru-Si approximants. J. Alloy. Compd..

[32] Kreiner G., Moguilnikov Y., Burkhardt U., Schäpers M. (2004). Hume-Rothery controlled formation of structurally complex alloy phases in the ternary Ga-Mg-Pd system. J. Non-Cryst. Solids..

[33] Tsai A.-P. (2004). A test of Hume-Rothery rules for stable quasicrystals. J. Non-Cryst. Solids..

[34] Clementi E., Raimondi D.L., Reinhardt W.P. (1967). Atomic Screening Constants from SCF Functions. II. Atoms with 37 to 86 Electrons. J. Chem. Phys..

[35] Lide D.R. (2006). Properties of solid. CRC Handbook of Chemistry and Physics..

[36] Capobianco C.J. (1998). Ruthenium solubility in hematite. Amer. Mineral..

[37] Wiley J.B., Poeppelmeier K.R. (1991). Reduction chemistry of platinum group metal perovskites. Mater. Res. Bull..

[38] Kaim W., Sarkar B. (2007). Mixed valency in ruthenium complexes-coordinative aspects. Coordin. Chem. Rev..

